# A double salt of iodo­bis­muthate: *cis*-aqua­iodidobis(1,10-phenanthroline)cobalt(II) tris­(1,10-phenanthroline)cobalt(II) *trans*-hexa-μ_2_-iodido-hexa­iodidotribismuthate(III)

**DOI:** 10.1107/S1600536811033460

**Published:** 2011-08-27

**Authors:** Jiongke Chen, Wenxiang Chai, Li Song, Yunyun Yang, Feng Niu

**Affiliations:** a College of Materials Science and Engineering, China Jiliang University, Hangzhou 310018, People’s Republic of China; b Department of Chemistry, Key Laboratory of Advanced Textile Materials and Manufacturing Technology of the Education Ministry, Zhejiang Sci-Tech University, Hangzhou 310018, People’s Republic of China

## Abstract

In the title complex, [Co(C_12_H_8_N_2_)_3_][CoI(C_12_H_8_N_2_)_2_(H_2_O)][Bi_3_I_12_], conventionally abbreviated [Co(phen)_3_][CoI(phen)_2_(H_2_O)][Bi_3_I_12_], where phen is 1,10-phenanthroline, the Co^II^ atom in one cation is coordinated by six N atoms from three phen ligands in an octa­hedral coordination while the Co^II^ atom in the other cation is coordinated octa­hedrally by four N atoms from two phen ligands, one water O atom and one I atom. In the anion, three Bi^III^ ions adopt an octa­hedral coordination constructed by six I^−^ ligands. The three BiI_6_ octa­hedra are fused together through *trans* face-sharing.

## Related literature

For related complexes containing the [Bi_3_I_12_]^3−^ trinuclear cluster anion, see: Geiser *et al.* (1990 ▶); Carmalt *et al.* (1995 ▶); Okrut & Feldmann (2006 ▶); Sharutin *et al.* (2009 ▶). For complexes containing the [Co(phen)_3_]^2+^ cation, see: Liu *et al.* (2003 ▶); Harding *et al.* (2008 ▶); Tershansy *et al.* (2005 ▶); Hanauer *et al.* (2008 ▶); Boys *et al.* (1984 ▶) and for those containing the [CoCl(phen)_2_(H_2_O)]^+^ cation, see: Arun Kumar *et al.* (2009 ▶); Zhong *et al.* (2006 ▶, 2007 ▶). For related halogenidoanti­mon­ates(III) and -bis­muthates(III) crystallizing in non-centrosymmetric space groups and their physical properties, see: Jozkow *et al.* (2001 ▶).
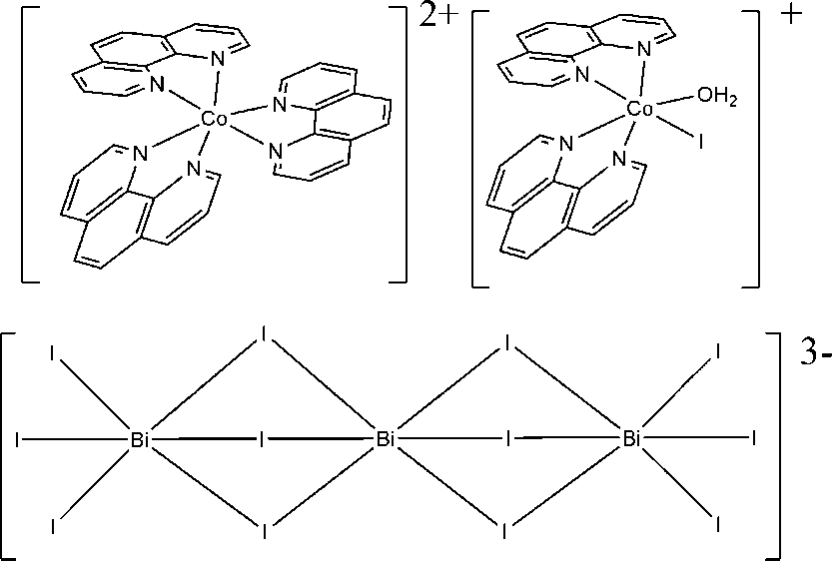


## Experimental

### 

#### Crystal data

[Co(C_12_H_8_N_2_)_3_][CoI(C_12_H_8_N_2_)_2_(H_2_O)][Bi_3_I_12_]
*M* *_r_* = 3313.6
Orthorhombic, 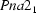

*a* = 35.188 (8) Å
*b* = 17.641 (4) Å
*c* = 12.793 (3) Å
*V* = 7941 (3) Å^3^
*Z* = 4
Mo *K*α radiation
μ = 12.13 mm^−1^
*T* = 293 K
0.30 × 0.24 × 0.20 mm

#### Data collection

Rigaku R-AXIS RAPID diffractometer
Absorption correction: multi-scan (*ABSCOR*; Higashi, 1995 ▶) *T*_min_ = 0.043, *T*_max_ = 0.088
58697 measured reflections
18072 independent reflections
16129 reflections with *I* > 2σ(*I*)
*R*_int_ = 0.037

#### Refinement

*R*[*F* ^2^ > 2σ(*F* ^2^)] = 0.037
*wR*(*F* ^2^) = 0.082
*S* = 1.04
18072 reflections
802 parameters
1 restraint
H-atom parameters constrained
Δρ_max_ = 1.29 e Å^−3^
Δρ_min_ = −2.13 e Å^−3^
Absolute structure: Flack (1983 ▶), 8549 Friedel pairs
Flack parameter: −0.021 (3)

### 

Data collection: *PROCESS-AUTO* (Rigaku, 1998 ▶); cell refinement: *PROCESS-AUTO*; data reduction: *CrystalStructure* (Rigaku/MSC, 2004 ▶); program(s) used to solve structure: *SHELXS97* (Sheldrick, 2008 ▶); program(s) used to refine structure: *SHELXL97* (Sheldrick, 2008 ▶); molecular graphics: *SHELXTL* (Sheldrick, 2008 ▶); software used to prepare material for publication: *SHELXTL*.

## Supplementary Material

Crystal structure: contains datablock(s) I, global. DOI: 10.1107/S1600536811033460/om2460sup1.cif

Structure factors: contains datablock(s) I. DOI: 10.1107/S1600536811033460/om2460Isup2.hkl

Additional supplementary materials:  crystallographic information; 3D view; checkCIF report

## Figures and Tables

**Table 1:** Selected bond lengths (Å)

Bi1—I1	2.8531 (10)
Bi1—I2	2.8901 (10)
Bi1—I3	2.9037 (10)
Bi1—I4	3.3444 (11)
Bi1—I6	3.3778 (9)
Bi1—I5	3.4186 (11)
Bi2—I9	3.0269 (10)
Bi2—I6	3.0523 (10)
Bi2—I8	3.0713 (9)
Bi2—I4	3.0955 (10)
Bi2—I5	3.0992 (10)
Bi2—I7	3.1756 (10)
Bi3—I10	2.9054 (11)
Bi3—I12	2.9135 (10)
Bi3—I11	2.9244 (10)
Bi3—I9	3.3139 (11)
Bi3—I7	3.3757 (10)
Bi3—I8	3.3829 (10)
I13—Co1	2.7815 (18)
Co1—N1	2.104 (8)
Co1—N4	2.128 (8)
Co1—N3	2.138 (9)
Co1—O1	2.154 (8)
Co1—N2	2.165 (9)
Co2—N7	2.124 (7)
Co2—N10	2.125 (7)
Co2—N9	2.126 (8)
Co2—N5	2.126 (8)
Co2—N8	2.130 (8)
Co2—N6	2.142 (8)

**Fig. 1.**
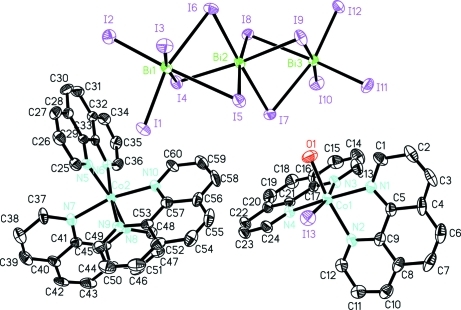
A view of the structure showing the atom-numbering scheme. Displacement ellipsoids are drawn at the 30% probabilithy level and H atoms are omitted for clarity.

**Fig. 2.**
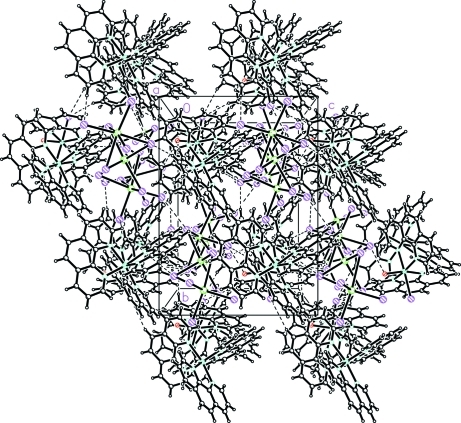
Packing diagram.

## supplementary crystallographic information

### Comment

Alkylammonium halogenoantimonates(III) and bismuthates(III) of general formula *R*_a_*M*_b_*X*_3 b+a_ (where *R* denotes organic cations, M—Sb(III), Bi(III), and *X*—Cl, Br, I) form a large group of crystals exhibiting interesting physical properties from their noncentrosymmetric space groups, *e.g.*, ferroelectricity (Jozkow *et al.*, 2001). Of them, *R*_3_*M*_2_*X*_9_ and *R*_5_*M*_2_*X*_11_ have been studied in detail for their phase transitions and physical properties. [Bi_3_I_12_]^3-^ is potentially chiral, but there is only one such example containing *cis*-[Bi_3_I_12_]^3-^ reported (Sharutin *et al.* 2009). In searching for other crystals in a noncentrosymmetric space group of halogenobismuthate(III), we employed a potentially optically active complex cation, *e.g.*, [Co(phen)_3_]^2-^ to synthesize a non-centrosymmetric compound. Here, we report one of those iodobismuthate(III) complexes, [Co(phen)_3_][CoI(phen)_2_(H_2_O)][Bi_3_I_12_], composed of the trinuclear Bi(III) cluster anion of *trans*-[Bi_3_I_12_]^3-^, and the cations containing cobalt(II) in a noncentrosymmetric space group.

In the title compound, three Bi atoms are all located in an octahedral environment of six I atoms, and three BiI_6_ octahedra are fused togather to form a *trans*-[Bi_3_I_12_]^3-^ trinuclear cluster by *trans* face-sharing through two pairs of three µ_2_-I atoms. In three BiI_6_ octahedra, the bond lengths of Bi—I ranged from 2.853 (1) Å to 3.419 (1) Å. All bond lengths are within commonly accepted values in the literature (Sharutin *et al.* 2009; Geiser *et al.*, 1990; Carmalt *et al.*, 1995; Okrut *et al.*, 2006). In the two cations of the title compound, one cobalt(II) is located in an octahedral environment constructed by six N atoms from three phen ligands to form a [Co(phen)_3_]^2+^ cation, and the other cobalt(II) is located in a distorted octahedral environment constructed by four N atoms from two phen ligands, one O atom from water and one I atom to form a cis-[CoI(phen)_2_(H_2_O)]^+^ cation. In the cation of [Co(phen)_3_]^2+^, the bond lengths of Co—N range from 2.124 (7) Å to 2.142 (8) Å, which are similar reported literature values (Liu *et al.*, 2003; Harding *et al.*, 2008; Tershansy *et al.* 2005; Hanauer *et al.* 2008; Boys *et al.* 1984). In the cation of [CoI(phen)_2_(H_2_O)]^+^, Co1—O1 = 2.154 (8) Å, Co1—I13 = 2.782 (2) Å, the bond lengths of Co—N ranged from 2.104 (8) Å to 2.165 (9) Å. This structure is similar to that of the reported [CoCl(phen)_2_(H_2_O)]^+^ cation (Arun Kumar *et al.*, 2009; Zhong *et al.*, 2007; Zhong *et al.*, 2006)

### Experimental

The title compound was synthesized by a solvothermal reaction of CoCl_2_ hexahydrate (48 mg, 0.2 mmol), BiI_3_ (180 mg, 0.3 mmol), KI (68 mg, 0.4 mmol) and 1,10-phenanthroline monohydrate (100 mg, 0.5 mmol) in 15 ml ethanol. The mixture was heated to 383 K at the rate of 20 K/h, and kept at this temperature for 2 days and then cooled to room temperature at the rate of 2 K/h. The red crystals were obtained in a yield of 47% (156 mg). Anal. Calc. for C_60_H_42_Bi_3_Co_2_I_13_N_10_O (%): C, 21.75; H, 1.28; N, 4.23; O, 0.48. Found: C, 21.47; H, 1.46; N, 4.52;O, 0.63. Crystals suitable for single-crystal X-ray diffraction were selected directly from the sample as prepared.

### Refinement

All hydrogen atoms attached to C were added at calculated positions and refined using a riding model (C-H = 0.93 Å). Due to the presence of Bi in the structure, those pertaining to the coordinated water O1 could not be found in the difference Fourier map and were not included in the model.

### Crystal data

**Table:**

[Co(C_12_H_8_N_2_)_3_][CoI(C_12_H_8_N_2_)_2_(H_2_O)][Bi_3_I_12_]	*F*(000) = 5880
*M**_r_* = 3313.6	*D*_x_ = 2.770 Mg m^−^^3^
Orthorhombic, *P**n**a*2_1_	Mo *K*α radiation, λ = 0.71075 Å
Hall symbol: P 2c -2n	Cell parameters from 3822 reflections
*a* = 35.188 (8) Å	θ = 2.1–27.5°
*b* = 17.641 (4) Å	µ = 12.13 mm^−^^1^
*c* = 12.793 (3) Å	*T* = 293 K
*V* = 7941 (3) Å^3^	Prism, red
*Z* = 4	0.30 × 0.24 × 0.20 mm

### Data collection

**Table:**

Rigaku R-AXIS RAPID diffractometer	18072 independent reflections
Radiation source: fine-focus sealed tube	16129 reflections with *I* > 2σ(*I*)
graphite	*R*_int_ = 0.037
Detector resolution: 14.6306 pixels mm^-1^	θ_max_ = 27.5°, θ_min_ = 2.1°
CCD_Profile_fitting scans	*h* = −45→45
Absorption correction: multi-scan (*ABSCOR*; Higashi, 1995)	*k* = −22→20
*T*_min_ = 0.043, *T*_max_ = 0.088	*l* = −16→16
58697 measured reflections	

### Refinement

**Table:**

Refinement on *F*^2^	Secondary atom site location: difference Fourier map
Least-squares matrix: full	Hydrogen site location: inferred from neighbouring sites
*R*[*F*^2^ > 2σ(*F*^2^)] = 0.037	H-atom parameters constrained
*wR*(*F*^2^) = 0.082	*w* = 1/[σ^2^(*F*_o_^2^) + (0.0228*P*)^2^] where *P* = (*F*_o_^2^ + 2*F*_c_^2^)/3
*S* = 1.04	(Δ/σ)_max_ = 0.003
18072 reflections	Δρ_max_ = 1.29 e Å^−^^3^
802 parameters	Δρ_min_ = −2.13 e Å^−^^3^
1 restraint	Absolute structure: Flack (1983), 8549 Friedel pairs
Primary atom site location: structure-invariant direct methods	Flack parameter: −0.021 (3)

### Special details

**Table:**

Geometry. All e.s.d.'s (except the e.s.d. in the dihedral angle between two l.s. planes) are estimated using the full covariance matrix. The cell e.s.d.'s are taken into account individually in the estimation of e.s.d.'s in distances, angles and torsion angles; correlations between e.s.d.'s in cell parameters are only used when they are defined by crystal symmetry. An approximate (isotropic) treatment of cell e.s.d.'s is used for estimating e.s.d.'s involving l.s. planes.
Refinement. Refinement of *F*^2^ against ALL reflections. The weighted *R*-factor *wR* and goodness of fit *S* are based on *F*^2^, conventional *R*-factors *R* are based on *F*, with *F* set to zero for negative *F*^2^. The threshold expression of *F*^2^ > σ(*F*^2^) is used only for calculating *R*-factors(gt) *etc*. and is not relevant to the choice of reflections for refinement. *R*-factors based on *F*^2^ are statistically about twice as large as those based on *F*, and *R*- factors based on ALL data will be even larger.

### Fractional atomic coordinates and isotropic or equivalent isotropic displacement parameters (Å^2^)

**Table:**

	*x*	*y*	*z*	*U*_iso_*/*U*_eq_	
Bi1	0.729444 (11)	0.41490 (2)	0.76524 (3)	0.04238 (8)	
Bi2	0.823474 (10)	0.27776 (2)	0.74040 (3)	0.04182 (8)	
Bi3	0.927594 (10)	0.16140 (2)	0.72011 (3)	0.04570 (9)	
I1	0.70619 (2)	0.45693 (5)	0.97088 (6)	0.0594 (2)	
I2	0.65702 (2)	0.37075 (5)	0.67782 (7)	0.0603 (2)	
I3	0.72529 (2)	0.56938 (4)	0.68774 (7)	0.0620 (2)	
I4	0.74462 (2)	0.23346 (4)	0.82856 (6)	0.05358 (18)	
I5	0.82014 (2)	0.43104 (4)	0.85842 (6)	0.05498 (18)	
I6	0.78032 (2)	0.35076 (4)	0.55956 (6)	0.05609 (18)	
I7	0.87167 (2)	0.20602 (5)	0.92549 (6)	0.05428 (18)	
I8	0.83795 (2)	0.12167 (4)	0.64181 (6)	0.04925 (16)	
I9	0.89658 (2)	0.32820 (5)	0.63692 (8)	0.0665 (2)	
I10	0.94555 (3)	0.01567 (5)	0.81368 (8)	0.0797 (3)	
I11	0.99598 (3)	0.23902 (6)	0.79367 (8)	0.0851 (3)	
I12	0.95859 (2)	0.12464 (5)	0.51510 (7)	0.0630 (2)	
I13	0.91231 (2)	0.48170 (5)	1.23063 (8)	0.0690 (2)	
Co1	0.94658 (4)	0.34209 (8)	1.19425 (11)	0.0480 (3)	
Co2	0.66931 (3)	0.28855 (7)	1.24912 (10)	0.0357 (3)	
O1	0.9183 (3)	0.3532 (5)	1.0458 (7)	0.073 (2)	
N1	0.9959 (2)	0.3921 (5)	1.1321 (7)	0.050 (2)	
N2	0.9809 (2)	0.3580 (5)	1.3329 (7)	0.047 (2)	
N3	0.9643 (2)	0.2297 (5)	1.1549 (7)	0.051 (2)	
N4	0.9038 (2)	0.2740 (5)	1.2648 (7)	0.048 (2)	
N5	0.6377 (2)	0.3360 (4)	1.1235 (6)	0.0387 (17)	
N6	0.6602 (2)	0.1921 (5)	1.1506 (6)	0.0382 (17)	
N7	0.6195 (2)	0.2741 (4)	1.3408 (7)	0.0427 (19)	
N8	0.6899 (2)	0.2230 (4)	1.3769 (6)	0.0388 (17)	
N9	0.6823 (2)	0.3941 (4)	1.3209 (6)	0.0388 (17)	
N10	0.7250 (2)	0.3095 (4)	1.1913 (6)	0.0395 (17)	
C1	1.0035 (4)	0.4052 (7)	1.0297 (9)	0.062 (3)	
H1	0.9864	0.3893	0.9789	0.075*	
C2	1.0369 (4)	0.4425 (7)	0.9999 (12)	0.069 (4)	
H2	1.0422	0.4496	0.9293	0.083*	
C3	1.0610 (4)	0.4677 (8)	1.0713 (12)	0.073 (4)	
H3	1.0820	0.4961	1.0511	0.087*	
C4	1.0551 (3)	0.4522 (6)	1.1748 (11)	0.060 (3)	
C5	1.0217 (3)	0.4127 (6)	1.2018 (9)	0.048 (2)	
C6	1.0792 (3)	0.4725 (7)	1.2596 (14)	0.080 (4)	
H6	1.1020	0.4968	1.2442	0.096*	
C7	1.0708 (4)	0.4582 (8)	1.3652 (14)	0.081 (5)	
H7	1.0879	0.4722	1.4172	0.097*	
C8	1.0364 (3)	0.4226 (6)	1.3917 (11)	0.058 (3)	
C9	1.0126 (3)	0.3984 (6)	1.3119 (9)	0.048 (2)	
C10	1.0258 (4)	0.4074 (7)	1.4943 (11)	0.064 (3)	
H10	1.0411	0.4239	1.5492	0.077*	
C11	0.9943 (4)	0.3698 (6)	1.5140 (10)	0.061 (3)	
H11	0.9869	0.3603	1.5825	0.073*	
C12	0.9719 (3)	0.3445 (6)	1.4301 (9)	0.051 (3)	
H12	0.9499	0.3171	1.4443	0.062*	
C13	0.9944 (4)	0.2074 (8)	1.0990 (12)	0.077 (4)	
H13	1.0113	0.2442	1.0757	0.093*	
C14	1.0017 (4)	0.1333 (9)	1.0741 (13)	0.090 (5)	
H14	1.0229	0.1205	1.0346	0.108*	
C15	0.9767 (4)	0.0775 (9)	1.1092 (12)	0.083 (4)	
H15	0.9811	0.0271	1.0918	0.100*	
C16	0.9466 (3)	0.0957 (7)	1.1674 (11)	0.063 (3)	
C17	0.9406 (3)	0.1761 (6)	1.1893 (8)	0.044 (2)	
C18	0.9197 (4)	0.0419 (7)	1.2025 (12)	0.076 (4)	
H18	0.9228	−0.0092	1.1869	0.092*	
C19	0.8883 (4)	0.0664 (8)	1.2610 (12)	0.079 (4)	
H19	0.8710	0.0306	1.2853	0.095*	
C20	0.8819 (3)	0.1462 (6)	1.2848 (9)	0.050 (2)	
C21	0.9077 (3)	0.1987 (5)	1.2484 (7)	0.040 (2)	
C22	0.8512 (3)	0.1718 (8)	1.3433 (9)	0.064 (3)	
H22	0.8336	0.1377	1.3706	0.076*	
C23	0.8474 (3)	0.2461 (8)	1.3595 (9)	0.064 (3)	
H23	0.8270	0.2646	1.3978	0.076*	
C24	0.8737 (3)	0.2943 (6)	1.3196 (9)	0.054 (3)	
H24	0.8703	0.3458	1.3319	0.065*	
C25	0.6257 (3)	0.4058 (6)	1.1125 (8)	0.046 (2)	
H25	0.6307	0.4407	1.1653	0.055*	
C26	0.6054 (3)	0.4299 (7)	1.0230 (9)	0.057 (3)	
H26	0.5979	0.4802	1.0160	0.068*	
C27	0.5969 (3)	0.3778 (7)	0.9469 (9)	0.058 (3)	
H27	0.5825	0.3924	0.8893	0.070*	
C28	0.6094 (3)	0.3047 (6)	0.9551 (7)	0.040 (2)	
C29	0.6294 (2)	0.2841 (5)	1.0462 (7)	0.0351 (19)	
C30	0.6035 (3)	0.2468 (8)	0.8777 (8)	0.060 (3)	
H30	0.5914	0.2599	0.8157	0.072*	
C31	0.6148 (3)	0.1740 (7)	0.8911 (8)	0.057 (3)	
H31	0.6091	0.1375	0.8411	0.069*	
C32	0.6356 (3)	0.1540 (6)	0.9819 (8)	0.046 (2)	
C33	0.6427 (2)	0.2095 (6)	1.0599 (7)	0.038 (2)	
C34	0.6498 (3)	0.0805 (6)	0.9990 (9)	0.057 (3)	
H34	0.6469	0.0433	0.9480	0.068*	
C35	0.6677 (3)	0.0638 (6)	1.0902 (10)	0.057 (3)	
H35	0.6765	0.0151	1.1037	0.069*	
C36	0.6726 (3)	0.1211 (6)	1.1621 (9)	0.050 (3)	
H36	0.6855	0.1094	1.2234	0.060*	
C37	0.5847 (3)	0.3036 (7)	1.3247 (11)	0.062 (3)	
H37	0.5796	0.3265	1.2607	0.074*	
C38	0.5558 (4)	0.3012 (7)	1.4008 (11)	0.068 (4)	
H38	0.5325	0.3247	1.3889	0.082*	
C39	0.5626 (3)	0.2635 (7)	1.4929 (10)	0.066 (4)	
H39	0.5438	0.2612	1.5437	0.079*	
C40	0.5980 (3)	0.2284 (6)	1.5112 (8)	0.051 (3)	
C41	0.6260 (3)	0.2371 (6)	1.4333 (7)	0.042 (2)	
C42	0.6072 (4)	0.1857 (7)	1.6013 (8)	0.060 (3)	
H42	0.5890	0.1796	1.6535	0.072*	
C43	0.6425 (5)	0.1528 (8)	1.6137 (9)	0.077 (4)	
H43	0.6473	0.1228	1.6720	0.092*	
C44	0.6716 (4)	0.1641 (7)	1.5385 (8)	0.058 (3)	
C45	0.6637 (3)	0.2072 (6)	1.4496 (7)	0.040 (2)	
C46	0.7092 (4)	0.1379 (7)	1.5523 (10)	0.068 (4)	
H46	0.7159	0.1101	1.6113	0.082*	
C47	0.7350 (4)	0.1540 (7)	1.4780 (9)	0.058 (3)	
H47	0.7598	0.1357	1.4835	0.069*	
C48	0.7247 (3)	0.1973 (7)	1.3949 (8)	0.052 (3)	
H48	0.7435	0.2099	1.3468	0.063*	
C49	0.6608 (3)	0.4361 (6)	1.3840 (9)	0.051 (3)	
H49	0.6364	0.4194	1.4002	0.062*	
C50	0.6736 (4)	0.5045 (7)	1.4270 (10)	0.066 (3)	
H50	0.6577	0.5331	1.4696	0.079*	
C51	0.7087 (4)	0.5284 (6)	1.4065 (9)	0.062 (3)	
H51	0.7171	0.5740	1.4345	0.074*	
C52	0.7326 (3)	0.4864 (6)	1.3446 (8)	0.054 (3)	
C53	0.7180 (3)	0.4188 (5)	1.3011 (7)	0.040 (2)	
C54	0.7714 (3)	0.5072 (7)	1.3148 (10)	0.062 (3)	
H54	0.7814	0.5526	1.3393	0.074*	
C55	0.7925 (3)	0.4640 (7)	1.2545 (12)	0.069 (4)	
H55	0.8175	0.4782	1.2410	0.083*	
C56	0.7786 (3)	0.3974 (7)	1.2103 (9)	0.053 (3)	
C57	0.7410 (3)	0.3753 (6)	1.2326 (8)	0.044 (2)	
C58	0.8006 (3)	0.3490 (8)	1.1439 (11)	0.072 (4)	
H58	0.8259	0.3598	1.1292	0.086*	
C59	0.7832 (4)	0.2867 (8)	1.1031 (10)	0.065 (3)	
H59	0.7971	0.2559	1.0579	0.078*	
C60	0.7461 (3)	0.2669 (6)	1.1253 (8)	0.052 (3)	
H60	0.7355	0.2240	1.0947	0.062*	

### Atomic displacement parameters (Å^2^)

**Table:**

	*U*^11^	*U*^22^	*U*^33^	*U*^12^	*U*^13^	*U*^23^
Bi1	0.04515 (18)	0.03708 (18)	0.04490 (19)	0.00082 (15)	0.01068 (16)	−0.00305 (16)
Bi2	0.04319 (18)	0.03795 (18)	0.04431 (19)	0.00065 (15)	0.00703 (15)	−0.00474 (16)
Bi3	0.03915 (17)	0.0468 (2)	0.0512 (2)	−0.00312 (16)	0.00139 (16)	−0.00271 (18)
I1	0.0661 (5)	0.0642 (5)	0.0480 (4)	0.0029 (4)	0.0210 (3)	−0.0015 (4)
I2	0.0588 (4)	0.0525 (4)	0.0695 (5)	−0.0107 (4)	−0.0018 (4)	−0.0047 (4)
I3	0.0789 (5)	0.0478 (4)	0.0593 (5)	−0.0110 (4)	0.0024 (4)	0.0031 (4)
I4	0.0535 (4)	0.0435 (4)	0.0636 (5)	−0.0051 (3)	0.0094 (3)	0.0048 (3)
I5	0.0583 (4)	0.0473 (4)	0.0593 (4)	−0.0091 (3)	0.0134 (3)	−0.0133 (3)
I6	0.0724 (5)	0.0548 (4)	0.0410 (4)	0.0145 (4)	0.0061 (3)	−0.0028 (3)
I7	0.0577 (4)	0.0608 (5)	0.0444 (4)	−0.0058 (4)	−0.0023 (3)	−0.0068 (3)
I8	0.0550 (4)	0.0424 (4)	0.0504 (4)	−0.0032 (3)	−0.0045 (3)	−0.0111 (3)
I9	0.0621 (5)	0.0546 (5)	0.0827 (6)	−0.0052 (4)	0.0257 (4)	0.0025 (4)
I10	0.0958 (7)	0.0570 (5)	0.0864 (7)	0.0046 (5)	−0.0075 (5)	0.0137 (5)
I11	0.0659 (5)	0.1084 (8)	0.0811 (7)	−0.0338 (5)	−0.0216 (5)	0.0048 (6)
I12	0.0617 (5)	0.0668 (5)	0.0605 (5)	−0.0092 (4)	0.0181 (4)	−0.0060 (4)
I13	0.0757 (5)	0.0556 (5)	0.0757 (6)	0.0044 (4)	−0.0007 (5)	−0.0070 (4)
Co1	0.0452 (7)	0.0521 (9)	0.0466 (8)	−0.0125 (7)	0.0058 (6)	−0.0026 (7)
Co2	0.0374 (6)	0.0384 (6)	0.0313 (6)	0.0002 (5)	0.0036 (5)	0.0018 (5)
O1	0.085 (6)	0.079 (6)	0.054 (5)	−0.016 (5)	−0.017 (4)	−0.004 (4)
N1	0.050 (5)	0.048 (5)	0.051 (5)	−0.018 (4)	0.004 (4)	−0.003 (4)
N2	0.037 (4)	0.052 (5)	0.051 (5)	0.001 (4)	0.007 (4)	0.012 (4)
N3	0.049 (5)	0.052 (5)	0.053 (5)	−0.003 (4)	0.014 (4)	0.003 (4)
N4	0.037 (4)	0.058 (5)	0.048 (5)	−0.003 (4)	0.005 (4)	−0.002 (4)
N5	0.042 (4)	0.039 (4)	0.036 (4)	0.006 (3)	0.007 (3)	0.008 (3)
N6	0.033 (4)	0.046 (5)	0.036 (4)	0.001 (3)	0.000 (3)	−0.002 (3)
N7	0.038 (4)	0.040 (5)	0.050 (5)	−0.005 (4)	0.010 (4)	0.011 (4)
N8	0.053 (5)	0.032 (4)	0.032 (4)	0.001 (4)	0.000 (3)	−0.005 (3)
N9	0.045 (4)	0.040 (4)	0.031 (4)	0.005 (4)	0.003 (3)	0.000 (3)
N10	0.038 (4)	0.038 (4)	0.042 (4)	−0.001 (3)	0.007 (3)	0.007 (4)
C1	0.077 (8)	0.055 (7)	0.055 (7)	−0.011 (6)	0.022 (6)	0.002 (6)
C2	0.066 (8)	0.059 (8)	0.083 (10)	0.006 (7)	0.033 (7)	0.017 (7)
C3	0.055 (7)	0.069 (9)	0.093 (11)	0.003 (7)	0.044 (7)	0.012 (8)
C4	0.033 (5)	0.033 (6)	0.114 (11)	−0.007 (4)	0.013 (6)	0.000 (6)
C5	0.040 (5)	0.042 (6)	0.062 (7)	−0.003 (4)	0.007 (5)	−0.002 (5)
C6	0.048 (6)	0.059 (8)	0.133 (14)	−0.020 (6)	0.004 (8)	−0.005 (9)
C7	0.055 (8)	0.054 (8)	0.134 (14)	−0.010 (6)	−0.030 (8)	−0.023 (9)
C8	0.050 (6)	0.040 (6)	0.082 (9)	−0.006 (5)	−0.016 (6)	−0.003 (6)
C9	0.041 (5)	0.033 (5)	0.070 (7)	0.002 (4)	−0.004 (5)	−0.008 (5)
C10	0.059 (7)	0.054 (8)	0.078 (9)	0.006 (6)	−0.016 (6)	−0.008 (6)
C11	0.072 (8)	0.051 (7)	0.059 (7)	0.018 (6)	−0.012 (6)	0.007 (6)
C12	0.051 (6)	0.045 (6)	0.058 (7)	0.006 (5)	−0.001 (5)	0.013 (5)
C13	0.053 (7)	0.084 (10)	0.095 (11)	0.015 (7)	0.021 (7)	0.014 (8)
C14	0.081 (10)	0.084 (11)	0.105 (13)	0.017 (9)	0.030 (9)	−0.009 (10)
C15	0.097 (11)	0.074 (10)	0.079 (10)	0.031 (9)	0.000 (8)	−0.018 (8)
C16	0.055 (7)	0.049 (7)	0.085 (9)	0.011 (6)	−0.005 (6)	0.002 (6)
C17	0.035 (5)	0.051 (6)	0.046 (5)	0.000 (4)	−0.003 (4)	0.005 (5)
C18	0.082 (9)	0.050 (7)	0.097 (11)	−0.015 (7)	0.004 (8)	−0.017 (7)
C19	0.080 (9)	0.068 (9)	0.088 (10)	−0.022 (7)	0.000 (8)	0.004 (8)
C20	0.046 (5)	0.050 (6)	0.053 (6)	−0.011 (5)	0.003 (4)	−0.006 (5)
C21	0.048 (5)	0.041 (5)	0.031 (5)	−0.005 (4)	0.000 (4)	−0.006 (4)
C22	0.056 (7)	0.084 (9)	0.051 (7)	−0.026 (6)	0.020 (5)	0.004 (6)
C23	0.056 (7)	0.078 (9)	0.057 (7)	−0.024 (6)	0.020 (6)	−0.019 (6)
C24	0.053 (6)	0.053 (7)	0.055 (7)	−0.013 (5)	0.017 (5)	−0.016 (5)
C25	0.049 (6)	0.040 (6)	0.050 (6)	0.009 (5)	0.008 (5)	0.003 (4)
C26	0.057 (7)	0.057 (7)	0.057 (7)	0.022 (6)	0.011 (5)	0.015 (6)
C27	0.046 (6)	0.077 (8)	0.052 (7)	0.014 (6)	−0.010 (5)	0.014 (6)
C28	0.036 (5)	0.048 (6)	0.038 (5)	0.000 (4)	0.001 (4)	0.011 (4)
C29	0.036 (4)	0.043 (5)	0.027 (4)	−0.005 (4)	0.005 (3)	0.005 (4)
C30	0.057 (7)	0.086 (9)	0.038 (6)	−0.019 (6)	−0.016 (5)	0.010 (6)
C31	0.062 (7)	0.068 (8)	0.041 (6)	−0.021 (6)	0.003 (5)	−0.008 (5)
C32	0.041 (5)	0.058 (7)	0.038 (5)	−0.004 (5)	0.002 (4)	−0.002 (5)
C33	0.026 (4)	0.045 (6)	0.041 (5)	−0.006 (4)	0.002 (4)	−0.003 (4)
C34	0.065 (7)	0.047 (7)	0.058 (7)	−0.014 (6)	0.007 (6)	−0.016 (5)
C35	0.057 (7)	0.038 (6)	0.077 (8)	0.009 (5)	−0.005 (6)	−0.003 (6)
C36	0.049 (6)	0.042 (6)	0.058 (7)	0.008 (5)	−0.012 (5)	0.004 (5)
C37	0.032 (5)	0.068 (8)	0.086 (9)	−0.001 (5)	0.018 (5)	0.019 (7)
C38	0.060 (7)	0.061 (8)	0.085 (10)	0.001 (6)	0.026 (7)	0.003 (7)
C39	0.065 (8)	0.060 (8)	0.074 (8)	−0.022 (6)	0.036 (6)	−0.007 (6)
C40	0.061 (7)	0.046 (6)	0.046 (6)	−0.019 (5)	0.020 (5)	−0.011 (5)
C41	0.050 (6)	0.043 (6)	0.034 (5)	−0.011 (5)	0.004 (4)	0.004 (4)
C42	0.087 (9)	0.062 (8)	0.031 (5)	−0.027 (7)	0.021 (5)	−0.006 (5)
C43	0.113 (12)	0.084 (10)	0.034 (6)	−0.021 (9)	0.010 (7)	0.011 (6)
C44	0.085 (8)	0.058 (7)	0.031 (5)	−0.019 (6)	−0.014 (5)	0.001 (5)
C45	0.051 (5)	0.039 (5)	0.029 (4)	−0.005 (4)	0.000 (4)	0.006 (4)
C46	0.089 (9)	0.050 (7)	0.065 (8)	0.005 (7)	−0.037 (7)	0.011 (6)
C47	0.067 (7)	0.055 (7)	0.051 (6)	0.003 (6)	−0.009 (6)	0.002 (5)
C48	0.043 (6)	0.066 (8)	0.048 (6)	0.006 (5)	−0.007 (4)	−0.003 (5)
C49	0.052 (6)	0.049 (6)	0.053 (6)	0.012 (5)	−0.005 (5)	−0.007 (5)
C50	0.095 (10)	0.045 (7)	0.057 (7)	0.013 (7)	0.008 (7)	−0.008 (6)
C51	0.099 (10)	0.037 (6)	0.050 (7)	0.000 (6)	−0.006 (6)	−0.006 (5)
C52	0.068 (7)	0.050 (7)	0.042 (6)	−0.012 (6)	−0.013 (5)	0.012 (5)
C53	0.049 (5)	0.037 (5)	0.034 (5)	−0.002 (4)	−0.005 (4)	0.003 (4)
C54	0.056 (7)	0.048 (7)	0.082 (9)	−0.011 (6)	−0.016 (6)	0.013 (6)
C55	0.034 (5)	0.073 (8)	0.101 (11)	−0.013 (5)	−0.009 (6)	0.028 (8)
C56	0.035 (5)	0.060 (7)	0.063 (7)	−0.013 (5)	−0.009 (5)	0.019 (6)
C57	0.047 (5)	0.047 (6)	0.039 (5)	0.003 (4)	−0.004 (4)	0.011 (4)
C58	0.036 (5)	0.097 (10)	0.083 (9)	0.001 (6)	0.027 (6)	0.021 (8)
C59	0.063 (8)	0.071 (9)	0.062 (8)	0.018 (7)	0.020 (6)	0.004 (6)
C60	0.056 (6)	0.053 (7)	0.046 (6)	−0.001 (5)	0.020 (5)	0.004 (5)

### Geometric parameters (Å, °)

**Table:**

Bi1—I1	2.8531 (10)	C15—H15	0.9300
Bi1—I2	2.8901 (10)	C16—C18	1.414 (17)
Bi1—I3	2.9037 (10)	C16—C17	1.461 (15)
Bi1—I4	3.3444 (11)	C17—C21	1.437 (13)
Bi1—I6	3.3778 (9)	C18—C19	1.403 (18)
Bi1—I5	3.4186 (11)	C18—H18	0.9300
Bi2—I9	3.0269 (10)	C19—C20	1.458 (16)
Bi2—I6	3.0523 (10)	C19—H19	0.9300
Bi2—I8	3.0713 (9)	C20—C21	1.379 (13)
Bi2—I4	3.0955 (10)	C20—C22	1.389 (15)
Bi2—I5	3.0992 (10)	C22—C23	1.334 (17)
Bi2—I7	3.1756 (10)	C22—H22	0.9300
Bi3—I10	2.9054 (11)	C23—C24	1.357 (14)
Bi3—I12	2.9135 (10)	C23—H23	0.9300
Bi3—I11	2.9244 (10)	C24—H24	0.9300
Bi3—I9	3.3139 (11)	C25—C26	1.415 (15)
Bi3—I7	3.3757 (10)	C25—H25	0.9300
Bi3—I8	3.3829 (10)	C26—C27	1.372 (16)
I13—Co1	2.7815 (18)	C26—H26	0.9300
Co1—N1	2.104 (8)	C27—C28	1.368 (15)
Co1—N4	2.128 (8)	C27—H27	0.9300
Co1—N3	2.138 (9)	C28—C29	1.410 (12)
Co1—O1	2.154 (8)	C28—C30	1.436 (15)
Co1—N2	2.165 (9)	C29—C33	1.406 (13)
Co2—N7	2.124 (7)	C30—C31	1.357 (16)
Co2—N10	2.125 (7)	C30—H30	0.9300
Co2—N9	2.126 (8)	C31—C32	1.416 (15)
Co2—N5	2.126 (8)	C31—H31	0.9300
Co2—N8	2.130 (8)	C32—C34	1.408 (15)
Co2—N6	2.142 (8)	C32—C33	1.420 (13)
N1—C5	1.322 (13)	C34—C35	1.358 (16)
N1—C1	1.357 (14)	C34—H34	0.9300
N2—C12	1.307 (13)	C35—C36	1.378 (15)
N2—C9	1.350 (12)	C35—H35	0.9300
N3—C17	1.336 (12)	C36—H36	0.9300
N3—C13	1.338 (14)	C37—C38	1.410 (15)
N4—C24	1.318 (12)	C37—H37	0.9300
N4—C21	1.351 (12)	C38—C39	1.375 (18)
N5—C25	1.309 (12)	C38—H38	0.9300
N5—C29	1.379 (12)	C39—C40	1.408 (17)
N6—C36	1.335 (12)	C39—H39	0.9300
N6—C33	1.348 (12)	C40—C41	1.410 (13)
N7—C37	1.346 (12)	C40—C42	1.416 (16)
N7—C41	1.371 (12)	C41—C45	1.444 (13)
N8—C48	1.324 (12)	C42—C43	1.381 (18)
N8—C45	1.339 (11)	C42—H42	0.9300
N9—C49	1.331 (12)	C43—C44	1.419 (17)
N9—C53	1.354 (12)	C43—H43	0.9300
N10—C60	1.353 (12)	C44—C45	1.396 (13)
N10—C57	1.395 (12)	C44—C46	1.413 (17)
C1—C2	1.399 (16)	C46—C47	1.346 (18)
C1—H1	0.9300	C46—H46	0.9300
C2—C3	1.325 (19)	C47—C48	1.360 (15)
C2—H2	0.9300	C47—H47	0.9300
C3—C4	1.367 (18)	C48—H48	0.9300
C3—H3	0.9300	C49—C50	1.401 (16)
C4—C5	1.410 (13)	C49—H49	0.9300
C4—C6	1.423 (18)	C50—C51	1.330 (17)
C5—C9	1.466 (15)	C50—H50	0.9300
C6—C7	1.41 (2)	C51—C52	1.373 (16)
C6—H6	0.9300	C51—H51	0.9300
C7—C8	1.404 (17)	C52—C53	1.414 (14)
C7—H7	0.9300	C52—C54	1.464 (16)
C8—C9	1.387 (15)	C53—C57	1.417 (13)
C8—C10	1.391 (18)	C54—C55	1.316 (18)
C10—C11	1.318 (17)	C54—H54	0.9300
C10—H10	0.9300	C55—C56	1.392 (16)
C11—C12	1.403 (16)	C55—H55	0.9300
C11—H11	0.9300	C56—C57	1.409 (13)
C12—H12	0.9300	C56—C58	1.432 (17)
C13—C14	1.369 (19)	C58—C59	1.362 (18)
C13—H13	0.9300	C58—H58	0.9300
C14—C15	1.39 (2)	C59—C60	1.383 (15)
C14—H14	0.9300	C59—H59	0.9300
C15—C16	1.333 (18)	C60—H60	0.9300
			
I1—Bi1—I2	100.02 (3)	C11—C12—H12	118.9
I1—Bi1—I3	93.24 (3)	N3—C13—C14	123.7 (13)
I2—Bi1—I3	94.38 (3)	N3—C13—H13	118.2
I1—Bi1—I4	94.08 (3)	C14—C13—H13	118.2
I2—Bi1—I4	88.66 (2)	C13—C14—C15	118.7 (13)
I3—Bi1—I4	171.47 (3)	C13—C14—H14	120.6
I1—Bi1—I6	163.21 (3)	C15—C14—H14	120.6
I2—Bi1—I6	94.34 (3)	C16—C15—C14	120.7 (14)
I3—Bi1—I6	94.30 (3)	C16—C15—H15	119.7
I4—Bi1—I6	77.50 (2)	C14—C15—H15	119.7
I1—Bi1—I5	85.67 (3)	C15—C16—C18	123.3 (13)
I2—Bi1—I5	168.70 (2)	C15—C16—C17	117.2 (12)
I3—Bi1—I5	95.04 (2)	C18—C16—C17	119.4 (11)
I4—Bi1—I5	81.16 (2)	N3—C17—C21	118.6 (9)
I6—Bi1—I5	78.74 (2)	N3—C17—C16	122.2 (10)
I9—Bi2—I6	88.12 (3)	C21—C17—C16	119.2 (9)
I9—Bi2—I8	86.73 (2)	C19—C18—C16	119.5 (12)
I6—Bi2—I8	98.60 (3)	C19—C18—H18	120.3
I9—Bi2—I4	174.50 (3)	C16—C18—H18	120.3
I6—Bi2—I4	86.37 (3)	C18—C19—C20	122.0 (12)
I8—Bi2—I4	94.13 (2)	C18—C19—H19	119.0
I9—Bi2—I5	89.36 (2)	C20—C19—H19	119.0
I6—Bi2—I5	88.99 (3)	C21—C20—C22	118.5 (10)
I8—Bi2—I5	171.34 (2)	C21—C20—C19	118.4 (10)
I4—Bi2—I5	90.51 (2)	C22—C20—C19	123.1 (11)
I9—Bi2—I7	89.39 (3)	N4—C21—C20	122.6 (9)
I6—Bi2—I7	177.41 (2)	N4—C21—C17	116.0 (9)
I8—Bi2—I7	81.97 (2)	C20—C21—C17	121.4 (9)
I4—Bi2—I7	96.11 (3)	C23—C22—C20	118.7 (10)
I5—Bi2—I7	90.27 (3)	C23—C22—H22	120.7
I10—Bi3—I12	95.31 (3)	C20—C22—H22	120.7
I10—Bi3—I11	95.90 (3)	C22—C23—C24	119.4 (11)
I12—Bi3—I11	94.93 (3)	C22—C23—H23	120.3
I10—Bi3—I9	171.74 (3)	C24—C23—H23	120.3
I12—Bi3—I9	91.82 (3)	N4—C24—C23	125.1 (11)
I11—Bi3—I9	87.62 (3)	N4—C24—H24	117.4
I10—Bi3—I7	90.71 (3)	C23—C24—H24	117.4
I12—Bi3—I7	166.11 (3)	N5—C25—C26	122.2 (10)
I11—Bi3—I7	96.89 (3)	N5—C25—H25	118.9
I9—Bi3—I7	81.43 (2)	C26—C25—H25	118.9
I10—Bi3—I8	98.14 (3)	C27—C26—C25	118.9 (10)
I12—Bi3—I8	92.08 (3)	C27—C26—H26	120.5
I11—Bi3—I8	163.65 (3)	C25—C26—H26	120.5
I9—Bi3—I8	77.40 (2)	C28—C27—C26	120.5 (10)
I7—Bi3—I8	74.65 (2)	C28—C27—H27	119.8
Bi2—I4—Bi1	79.24 (2)	C26—C27—H27	119.8
Bi2—I5—Bi1	78.040 (19)	C27—C28—C29	117.9 (10)
Bi2—I6—Bi1	79.31 (2)	C27—C28—C30	124.8 (10)
Bi2—I7—Bi3	79.86 (2)	C29—C28—C30	117.3 (9)
Bi2—I8—Bi3	81.218 (19)	N5—C29—C33	117.5 (8)
Bi2—I9—Bi3	83.02 (2)	N5—C29—C28	121.8 (9)
N1—Co1—N4	168.9 (4)	C33—C29—C28	120.6 (9)
N1—Co1—N3	93.4 (3)	C31—C30—C28	122.9 (10)
N4—Co1—N3	77.4 (3)	C31—C30—H30	118.5
N1—Co1—O1	90.6 (4)	C28—C30—H30	118.5
N4—Co1—O1	95.6 (3)	C30—C31—C32	119.4 (10)
N3—Co1—O1	90.6 (4)	C30—C31—H31	120.3
N1—Co1—N2	78.1 (3)	C32—C31—H31	120.3
N4—Co1—N2	97.0 (3)	C34—C32—C31	122.7 (10)
N3—Co1—N2	98.7 (3)	C34—C32—C33	117.6 (10)
O1—Co1—N2	165.7 (3)	C31—C32—C33	119.7 (10)
N1—Co1—I13	92.8 (3)	N6—C33—C29	118.1 (8)
N4—Co1—I13	97.0 (2)	N6—C33—C32	121.9 (9)
N3—Co1—I13	170.6 (3)	C29—C33—C32	119.9 (9)
O1—Co1—I13	82.3 (3)	C35—C34—C32	119.8 (10)
N2—Co1—I13	89.4 (2)	C35—C34—H34	120.1
N7—Co2—N10	166.7 (3)	C32—C34—H34	120.1
N7—Co2—N9	92.5 (3)	C34—C35—C36	118.3 (10)
N10—Co2—N9	78.5 (3)	C34—C35—H35	120.9
N7—Co2—N5	91.9 (3)	C36—C35—H35	120.9
N10—Co2—N5	98.7 (3)	N6—C36—C35	125.0 (10)
N9—Co2—N5	95.4 (3)	N6—C36—H36	117.5
N7—Co2—N8	78.0 (3)	C35—C36—H36	117.5
N10—Co2—N8	92.7 (3)	N7—C37—C38	122.7 (12)
N9—Co2—N8	94.1 (3)	N7—C37—H37	118.7
N5—Co2—N8	166.5 (3)	C38—C37—H37	118.7
N7—Co2—N6	96.1 (3)	C39—C38—C37	118.7 (12)
N10—Co2—N6	94.1 (3)	C39—C38—H38	120.7
N9—Co2—N6	169.2 (3)	C37—C38—H38	120.7
N5—Co2—N6	77.8 (3)	C38—C39—C40	120.7 (10)
N8—Co2—N6	94.1 (3)	C38—C39—H39	119.7
C5—N1—C1	118.1 (10)	C40—C39—H39	119.7
C5—N1—Co1	115.2 (7)	C39—C40—C41	116.9 (11)
C1—N1—Co1	126.7 (8)	C39—C40—C42	124.9 (10)
C12—N2—C9	119.1 (10)	C41—C40—C42	118.2 (11)
C12—N2—Co1	128.4 (7)	N7—C41—C40	123.0 (10)
C9—N2—Co1	111.6 (7)	N7—C41—C45	116.8 (8)
C17—N3—C13	117.5 (10)	C40—C41—C45	120.1 (9)
C17—N3—Co1	113.4 (7)	C43—C42—C40	121.5 (10)
C13—N3—Co1	129.0 (9)	C43—C42—H42	119.2
C24—N4—C21	115.7 (9)	C40—C42—H42	119.2
C24—N4—Co1	129.7 (8)	C42—C43—C44	120.8 (12)
C21—N4—Co1	114.6 (6)	C42—C43—H43	119.6
C25—N5—C29	118.7 (9)	C44—C43—H43	119.6
C25—N5—Co2	128.3 (7)	C45—C44—C46	117.8 (11)
C29—N5—Co2	113.0 (6)	C45—C44—C43	119.1 (12)
C36—N6—C33	117.3 (9)	C46—C44—C43	123.0 (12)
C36—N6—Co2	129.1 (7)	N8—C45—C44	122.8 (10)
C33—N6—Co2	113.2 (6)	N8—C45—C41	117.2 (8)
C37—N7—C41	117.9 (9)	C44—C45—C41	119.9 (9)
C37—N7—Co2	128.3 (7)	C47—C46—C44	118.3 (11)
C41—N7—Co2	113.3 (6)	C47—C46—H46	120.8
C48—N8—C45	116.4 (9)	C44—C46—H46	120.8
C48—N8—Co2	129.3 (7)	C46—C47—C48	119.4 (12)
C45—N8—Co2	114.3 (6)	C46—C47—H47	120.3
C49—N9—C53	117.5 (9)	C48—C47—H47	120.3
C49—N9—Co2	128.9 (7)	N8—C48—C47	125.1 (11)
C53—N9—Co2	113.6 (6)	N8—C48—H48	117.4
C60—N10—C57	118.5 (8)	C47—C48—H48	117.4
C60—N10—Co2	128.8 (7)	N9—C49—C50	122.3 (11)
C57—N10—Co2	112.6 (6)	N9—C49—H49	118.8
N1—C1—C2	120.5 (13)	C50—C49—H49	118.8
N1—C1—H1	119.8	C51—C50—C49	119.6 (11)
C2—C1—H1	119.8	C51—C50—H50	120.2
C3—C2—C1	120.6 (13)	C49—C50—H50	120.2
C3—C2—H2	119.7	C50—C51—C52	120.8 (11)
C1—C2—H2	119.7	C50—C51—H51	119.6
C2—C3—C4	120.2 (12)	C52—C51—H51	119.6
C2—C3—H3	119.9	C51—C52—C53	117.3 (11)
C4—C3—H3	119.9	C51—C52—C54	125.9 (11)
C3—C4—C5	117.5 (12)	C53—C52—C54	116.7 (11)
C3—C4—C6	126.7 (12)	N9—C53—C52	122.4 (10)
C5—C4—C6	115.8 (12)	N9—C53—C57	118.1 (9)
N1—C5—C4	122.9 (11)	C52—C53—C57	119.5 (10)
N1—C5—C9	116.8 (9)	C55—C54—C52	122.3 (11)
C4—C5—C9	120.1 (11)	C55—C54—H54	118.8
C7—C6—C4	124.2 (11)	C52—C54—H54	118.8
C7—C6—H6	117.9	C54—C55—C56	121.8 (11)
C4—C6—H6	117.9	C54—C55—H55	119.1
C8—C7—C6	119.6 (12)	C56—C55—H55	119.1
C8—C7—H7	120.2	C55—C56—C57	118.8 (11)
C6—C7—H7	120.2	C55—C56—C58	123.6 (10)
C9—C8—C10	118.3 (11)	C57—C56—C58	117.6 (10)
C9—C8—C7	118.7 (13)	N10—C57—C56	122.2 (10)
C10—C8—C7	123.0 (12)	N10—C57—C53	117.0 (8)
N2—C9—C8	121.0 (11)	C56—C57—C53	120.8 (10)
N2—C9—C5	117.5 (9)	C59—C58—C56	117.8 (10)
C8—C9—C5	121.5 (10)	C59—C58—H58	121.1
C11—C10—C8	120.2 (12)	C56—C58—H58	121.1
C11—C10—H10	119.9	C58—C59—C60	123.3 (11)
C8—C10—H10	119.9	C58—C59—H59	118.4
C10—C11—C12	119.2 (12)	C60—C59—H59	118.4
C10—C11—H11	120.4	N10—C60—C59	120.5 (11)
C12—C11—H11	120.4	N10—C60—H60	119.7
N2—C12—C11	122.2 (11)	C59—C60—H60	119.7
N2—C12—H12	118.9		

## References

[bb1] Arun Kumar, K., Dayalan, A. & SethuSankar, K. (2009). *Acta Cryst.* E**65**, m1300–m1301. 10.1107/S1600536809038422 PMC2971197 21578066

[bb2] Boys, D., Escobar, C. & Wittke, O. (1984). *Acta Cryst.* C**40**, 1359–1362.

[bb3] Carmalt, C. J., Farrugia, L. J. & Norman, N. C. (1995). *Z. Anorg. Allg. Chem.* **621**, 47–56.

[bb4] Flack, H. D. (1983). *Acta Cryst.* A**39**, 876–881.

[bb5] Geiser, U., Wade, E., Wang, H. H. & Williams, J. M. (1990). *Acta Cryst.* C**46**, 1547–1549.

[bb6] Hanauer, M., Neshat, A. & Bigioni, T. P. (2008). *Acta Cryst.* C**64**, m111–m113. 10.1107/S0108270108001972 18322319

[bb7] Harding, D. J., Harding, P. & Adams, H. (2008). *Acta Cryst.* E**64**, m1538. 10.1107/S1600536808036611 PMC2959989 21581152

[bb8] Higashi, T. (1995). *ABSCOR* Rigaku Corporation, Tokyo, Japan.

[bb9] Jozkow, J., Jakubas, R., Bator, G. & Pietraszko, A. (2001). *J. Chem. Phys.* **114**, 7239–7246.

[bb10] Liu, Y., Xu, D.-J. & Hung, C.-H. (2003). *Acta Cryst.* E**59**, m297–m299.

[bb11] Okrut, A. & Feldmann, C. (2006). *Z. Anorg. Allg. Chem.* **632**, 409–412.

[bb12] Rigaku (1998). *PROCESS-AUTO* Rigaku Corporation, Tokyo, Japan.

[bb13] Rigaku/MSC (2004). *CrystalStructure* Rigaku/MSC, The Woodlands, Texas, USA.

[bb14] Sharutin, V. V., Egorova, I. V., Klepikov, N. N., Boyarkina, E. A. & Sharutina, O. K. (2009). *Russ. J. Inorg. Chem.* **54**, 52–68.

[bb15] Sheldrick, G. M. (2008). *Acta Cryst.* A**64**, 112–122. 10.1107/S0108767307043930 18156677

[bb16] Tershansy, M. A., Goforth, A. M., Smith, M. D., Peterson Jr, L. R. & zur Loye, H.-C. (2005). *Acta Cryst.* E**61**, m1680–m1681.

[bb17] Zhong, H., Zeng, X.-R., Liu, Y.-Q. & Luo, Q.-Y. (2006). *Acta Cryst.* E**62**, m2925–m2927.

[bb18] Zhong, H., Zeng, X.-R. & Luo, Q.-Y. (2007). *Acta Cryst.* E**63**, m221–m223.

